# Supplementary material for: Detection and Genomic Characterization of Canine Circovirus in Iran
Source: Animals (Basel). 2022 Feb 17;12(4):507. doi: 10.3390/ani12040507 (PMC8868521; doi:10.3390/ani12040507)
Supplement: Supplementary file 1 [file animals-12-00507-s001.zip › animals-1519987-supplementary.pdf]

Table S1: Nucleotide and amino acid observed capsid sequences of Iranian CaCVs compared to CaCVs selected from phylogenetic groups from Table 2. Non-synonymous mutations in Iranian CaCVs are bolded. Highlighted in grey putative genotype-specific aa residues [34].

	Aa residue on capsid	13	23	50	83	103	123	148	178	231	234
Phylogenetic group	nt position	1890-1892	1860-1862	1779-1781	1680-1682	1620-1622	1560-1562	1485-1487	1395-1387	1236-1238	1227-1229
1	ITA/2013/dog/TE6685/1-13	S (GCT)	R (CCT)	V (TAC)	T (AGT)	R (CCT)	L (AAG)	I (TAT)	S (CGA)	M (CAT)	M (CAT)
2	CHN/2015/dog/WM48B6	R (TCT)	R (TCT)	V (CAC)	T (CGT)	T (CGT)	L (CAA)	N (ATT)	S (ACT)	M (CAT)	M (CAT)
3	CHN/2014/dog/JZ98	R (TCT)	R (CCT)	V (GAC)	T (TGT)	R (CCT)	L (TAG)	T (TGT)	A (TGC)	M (CAT)	M (CAT)
4	VNM/2017/dog/VN_1	R (CCT)	R (CCT)	V (CAC)	A (TGC)	R (TCT)	L (CAG)	I (TAT)	S (AGA)	M (CAT)	M (CAT)
5	ITA/2017/wolf/449	R (CCG)	R (CCT)	I (TAT)	V (TAC)	R (CCT)	L (AAG)	I (TAT)	T (TGT)	M (CAT)	M (CAT)
6	IRN/2019/Dog/292	S (ACT)	R (CCT)	V (CAC)	T (TGT)	R (TCT)	L (GAG)	**R (TCT)**	S (TGA)	M (CAT)	M (CAT)
	IRN/2019/Dog/298	S (ACT)	**K (CTT)**	V (CAC)	**I (TAT)**	R (TCT)	L (GAG)	**R (TCT)**	A (TGT)	M (CAT)	M (CAT)
	IRN/2019/Dog/308	**T (AGT)**	R (CCT)	V (CAC)	T (TGT)	R (TCT)	**I (GAT)**	**R (TCT)**	**Q (TTG)**	M (CAT)	M (CAT)
	IRN/2019/Dog/496	**T (AGT)**	R (CCT)	V (CAC)	T (TGT)	R (TCT)	L (GAG)	**R (TCT)**	T (TGT)	M (CAT)	M (CAT)
	IRN/2019/Dog/497	S (ACT)	R (CCT)	V (CAC)	T (TGT)	R (TCT)	L (GAG)	**R (TCT)**	S (TGA)	**I (TAT)**	**L (CAG)**
	IRN/2019/Dog/498	**T (AGT)**	R (CCT)	**M (CAT)**	T (TGT)	**K (CTT)**	L (GAG)	**R (TCT)**	A (TGT)	M (CAT)	M (CAT)
UCD3-487	USA/2011/dog/UCD3-478	R (CCG)	R (TCT)	V (TAC)	T (TGT)	R (CCT)	L (AAG)	I (TAT)	F (AAA)	M (CAT)	M (CAT)

Table S2: Nucleotide and amino acid observed replicase sequences of Iranian CaCVs compared to CaCVs selected from phylogenetic groups from Table 2. Non-synonymous mutations in Iranian CaCVs are bolded.

Phylogenetic group	Aa residue on replicase	8	10	32	37	55	69	72	73	75	76	77	78	106	132	140	142	150	174	257	269	282	300
	nt position	22-24	28-30	94-96	109-111	163-165	205-207	214-216	217-219	223-225	226-228	229-231	232-234	316-318	394-396	418-420	424-426	448-450	520-522	769-771	805-807	844-846	898-900
1	ITA/2013/dog/TE6685/1-13	Q (CAG)	V (GTC)	E (GAG)	V (GTG)	E (GAG)	K (AAG)	R (CGT)	M (ATG)	A (GCC)	L (CTG)	K (AAG)	A (GCA)	V (GTA	T (ACG)	S (TCT)	F (TTT)	S (TCG)	E (GAG)	N (AAT)	S (TCT)	K (AAG)	M (ATG)
2	CHN/2015/dog/WM48B6	Q (CAG)	G (GGC)	A (GCG)	V (GTG)	E (GAG)	N (AAT)	R (CGG)	M (ATG)	A (GCC)	L (TTG)	K (AAG)	A (GCT)	L (TTG)	T (ACG)	S (TCT)	Y (TAT)	S (TCG)	E (GAA)	N (AAT)	G (GGG)	K (AAG)	M (ATG)
3	CHN/2014/dog/JZ98	Q (CAG)	G (GGC)	E (GAA)	V (GTG)	E (GAG)	N (AAT)	R (CGG)	M (ATG)	A (GCC)	I (ATT)	K (AAG)	A (GCA)	I (ATT)	T (ACT)	A (GCT)	Y (TAT)	S (TCG)	E (GAG)	N (AAT)	G (GGG)	K (AAA)	M (ATG)
4	VNM/2017/dog/VN_1	Q (CAG)	V (GTC)	E (GAG)	V (GTG)	E (GAG)	K (AAG)	R (CGT)	M (ATG)	A (GCC)	L (CTG)	K (AAG)	A (GCA)	I (ATA)	T (ACG)	A (GCT)	Y (TAT)	S (TCG)	E (GAA)	N (AAT)	G (GGG)	K (AAA)	M (ATG)
5	ITA/2017/wolf/449	Q (CAA)	V (GTC)	A (GCG)	V (GTG)	E (GAG)	K (AAG)	R (CGT)	M (ATG)	A (GCT)	L (CTG)	K (AAG)	A (GCA)	I (ATC)	T (ACG)	A (GCT)	H (CAC)	S (TCG)	N (AAC)	N (AAT)	G (GGT)	T (ACT)	R (CGG)
6	IRN/2019/Dog/292	Q (CAG)	G (GGC)	**D (GAT)**	V (GTG)	E (GAG)	**Q (CAG)**	R (CGA)	M (ATG)	**I (ATC)**	L (CTC)	K (AAG)	A (GCA)	I (ATT)	T (ACC)	A (GCT)	Y (TAT)	S (TCG)	E (GAA)	N (AAT)	S (AGT)	K (AAA)	M (ATG)
	IRN/2019/Dog/298	**K (AAG)**	**A (GCC)**	**D (GAT)**	V (GTG)	E (GAG)	**Q (CAG)**	R (CGA)	M (ATG)	A (GCT)	**I (ATC)**	K (AAG)	A (GCA)	I (ATT)	T (ACC)	A (GCT)	Y (TAT)	S (TCG)	E (GAA)	**K (AAG)**	S (TCA)	**S (AGT)**	**L (CTG)**
	IRN/2019/Dog/308	Q (CAG)	V (GTC)	**D (GAT)**	V (GTG)	**A (GCG)**	**Q (CAG)**	R (CGA)	M (ATG)	A (GCT)	L (CTG)	**R (AGA)**	A (GCA)	I (ATT)	T (ACC)	**G (GGT)**	Y (TAT)	**A GCG)**	E (GAA)	N (AAT)	**C (TGT)**	K (AAA)	M (ATG)
	IRN/2019/Dog/496	**K (AAG)**	**A (GCC)**	E (GAG)	V (GTG)	E (GAG)	**R (CGA)**	R (CGG)	M (ATG)	A (GCT)	I (ATT)	K (AAA)	**G (GGC)**	I (ATT)	T (ACG)	A (GCT)	Y (TAT)	S (TCG)	**Q (CAA)**	N (AAT)	S (TCA)	**S (AGT)**	**L (CTG)**
	IRN/2019/Dog/497	Q (CAG)	G (GGC)	**D (GAT)**	**G (GGG)**	E (GAG)	**Q (CAG)**	R (CGT)	M (ATG)	A (GCT)	L (CTC)	K (AAG)	A (GCA)	**L (TTG)**	**S (TCC)**	A (GCT)	**F (TTT)**	S (TCG)	**Q (CAA)**	N (AAT)	S (AGT)	K (AAA)	M (ATG)
	IRN/2019/Dog/498	**K (AAG)**	**A (GCC)**	E (GAG)	V (GTG)	E (GAG)	**Q (CAG)**	**Q (CAG)**	**V (GTG)**	A (GCG)	L (CTA)	K (AAG)	A (GCA)	I (ATT)	T (ACG)	A (GCT)	Y (TAT)	S (TCG)	**Q (CAA)**	N (AAT)	S (TCA)	**S (AGT)**	**L (CTG)**
UCD3-487	USA/2011/dog/UCD3-478	H (CAT)	V (GTC)	E (GAG)	V (GTG)	E (GAG)	K (AAG)	R (CGT)	M (ATG)	A (GCC)	L (CTG)	K (AAG)	A (GCA)	I (ATA)	T (ACG)	A (GCT)	H (CAC)	S (TCG)	H (CAC)	N (AAT)	S (AGT)	T (ACT)	M (ATG)
